# *catena*-Poly[[bis­(*O*,*O*′-diisopropyl dithio­phosphato-κ^2^ *S*,*S*′)nickel(II)]-μ-bis­(4-pyridylmethyl­ene)diazane-κ^2^ *N*:*N*′]

**DOI:** 10.1107/S1600536809043505

**Published:** 2009-10-28

**Authors:** Erick Berdugo, Edward R. T. Tiekink

**Affiliations:** a Department of Chemistry, Texas A&M University, College Station, Texas 77842-3012, USA; b Department of Chemistry, University of Malaya, 50603 Kuala Lumpur, Malaysia

## Abstract

The Ni atom in the title linear supra­molecular polymer, [Ni(C_6_H_14_O_2_PS_2_)_2_(C_12_H_10_N_4_)]_*n*_, exists within a *trans*-N_2_S_4_ octa­hedral donor set defined by two symmetrically coordinating dithio­phosphate ligands and pyridine N atoms derived from two bridging bis­(4-pyridylmethyl­ene)diazane ligands. The Ni atom lies on a centre of inversion and the bis­(4-pyridylmethyl­ene)diazane ligand is also disposed about a centre of inversion. The chains are arranged into layers sustained by C—H⋯S contacts and inter­digitate with neighbouring layers, forming the crystal structure.

## Related literature

For background to supra­molecular polymers of metal dithio­phosphates, see: Lai & Tiekink (2004 ▶); Chen *et al.* (2006 ▶); Aragoni *et al.* (2007 ▶). For a related *iso*-butyl structure and the synthesis, see: Berdugo & Tiekink (2008 ▶).
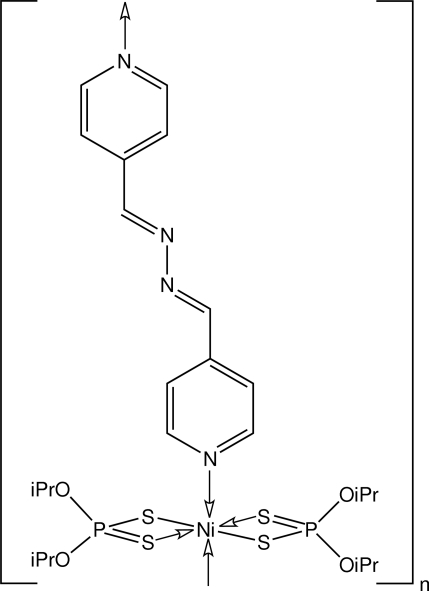


## Experimental

### 

#### Crystal data

[Ni(C_6_H_14_O_2_PS_2_)_2_(C_12_H_10_N_4_)]
*M* *_r_* = 695.47
Triclinic, 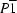

*a* = 8.661 (2) Å
*b* = 8.753 (2) Å
*c* = 11.159 (3) Å
α = 88.110 (8)°
β = 81.502 (7)°
γ = 89.813 (10)°
*V* = 836.2 (4) Å^3^
*Z* = 1
Mo *K*α radiation
μ = 0.96 mm^−1^
*T* = 98 K
0.50 × 0.08 × 0.05 mm

#### Data collection

Rigaku AFC12K/SATURN724 diffractometer
Absorption correction: multi-scan (*ABSCOR*; Higashi, 1995 ▶) *T*_min_ = 0.794, *T*_max_ = 1
7552 measured reflections
3810 independent reflections
3555 reflections with *I* > 2σ(*I*)
*R*_int_ = 0.036

#### Refinement

*R*[*F* ^2^ > 2σ(*F* ^2^)] = 0.043
*wR*(*F* ^2^) = 0.111
*S* = 1.09
3810 reflections
178 parameters
H-atom parameters constrained
Δρ_max_ = 0.63 e Å^−3^
Δρ_min_ = −0.70 e Å^−3^

### 

Data collection: *CrystalClear* (Rigaku/MSC 2005 ▶); cell refinement: *CrystalClear*; data reduction: *CrystalClear*; program(s) used to solve structure: *SHELXS97* (Sheldrick, 2008 ▶); program(s) used to refine structure: *SHELXL97* (Sheldrick, 2008 ▶); molecular graphics: *DIAMOND* (Brandenburg, 2006 ▶); software used to prepare material for publication: *SHELXL97*.

## Supplementary Material

Crystal structure: contains datablocks global, I. DOI: 10.1107/S1600536809043505/hb5163sup1.cif

Structure factors: contains datablocks I. DOI: 10.1107/S1600536809043505/hb5163Isup2.hkl

Additional supplementary materials:  crystallographic information; 3D view; checkCIF report

## Figures and Tables

**Table:** 

Ni—S1	2.4827 (7)
Ni—S2	2.4835 (7)
Ni—N1	2.1051 (19)

**Table:** 

S1—Ni—S2	82.46 (2)

**Table 2:** Hydrogen-bond geometry (Å, °)

*D*—H⋯*A*	*D*—H	H⋯*A*	*D*⋯*A*	*D*—H⋯*A*
C8—H8⋯S2^i^	0.95	2.77	3.694 (3)	164

Symmetry code: (i) 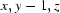.

**Fig. 1.**
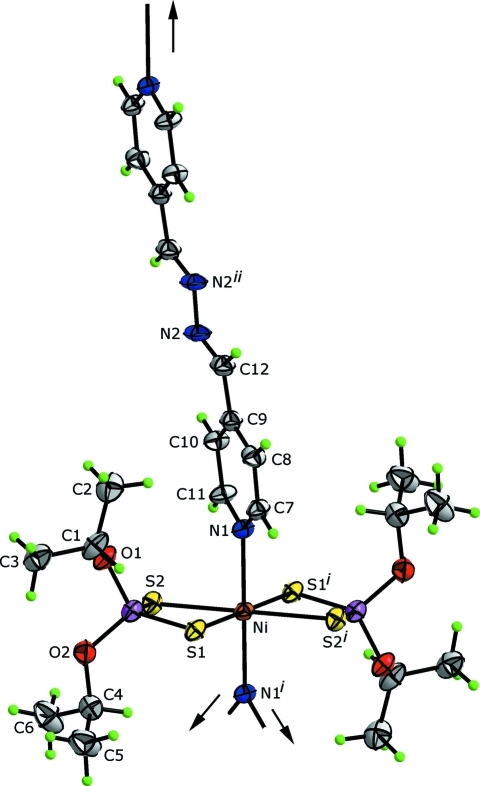
The asymmetric unit of (I) extended to show the Ni atom coordination geometry and a full molecule of the 4-pyridineazine ligand, showing displacement ellipsoids at the 50% probability level. Symmetry operation i: 1 - x, 1 - y, 1 - z and ii: 1 + x, 1 + y, -1 + z.

**Fig. 2.**
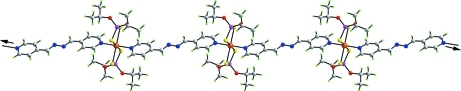
Supramolecular chain in (I) with base vector [111].

**Fig. 3.**
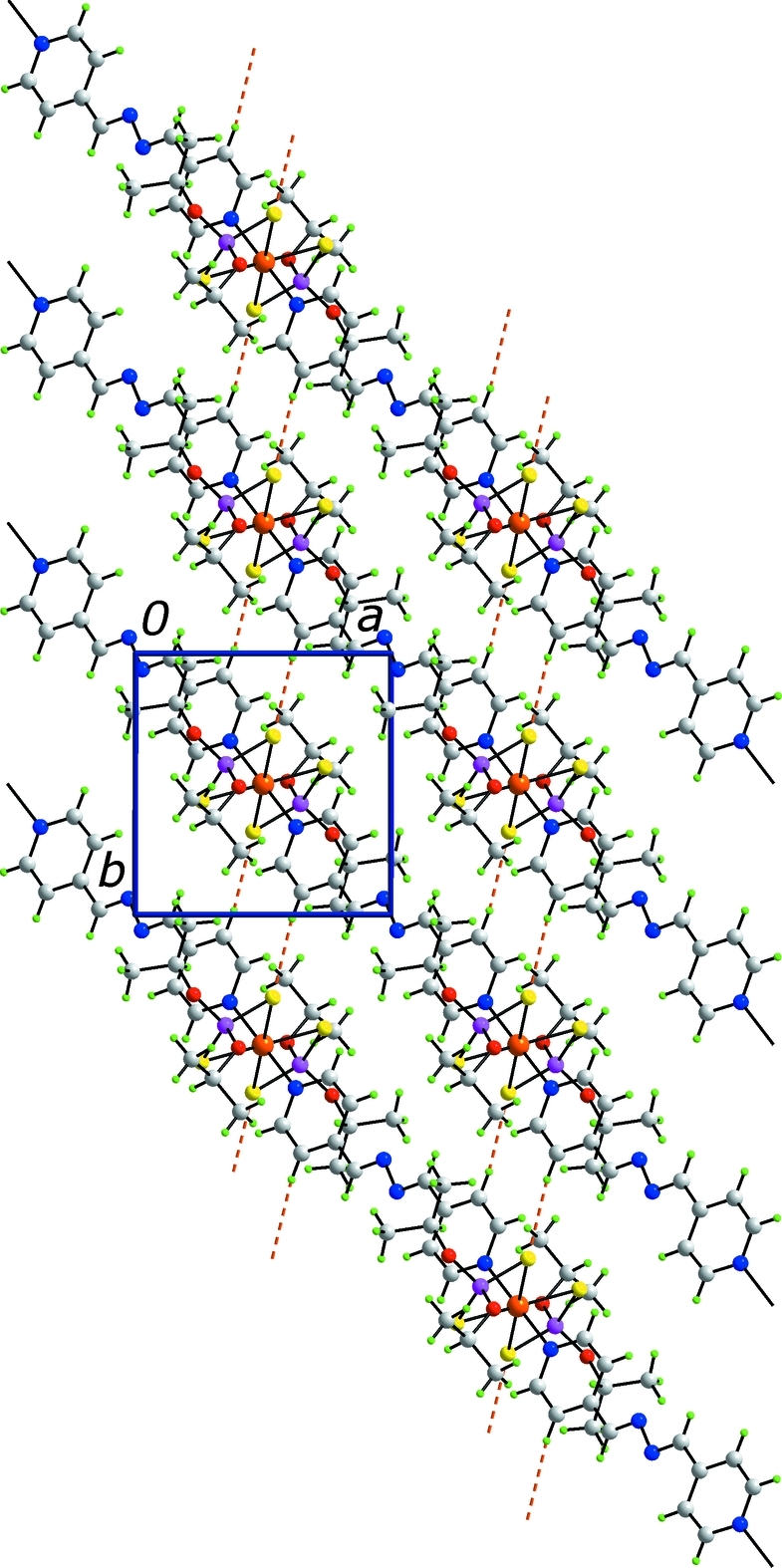
Layers of supramolecular chains mediated by C—H···S contacts (orange dashed lines), viewed in projection down the c axis.

**Fig. 4.**
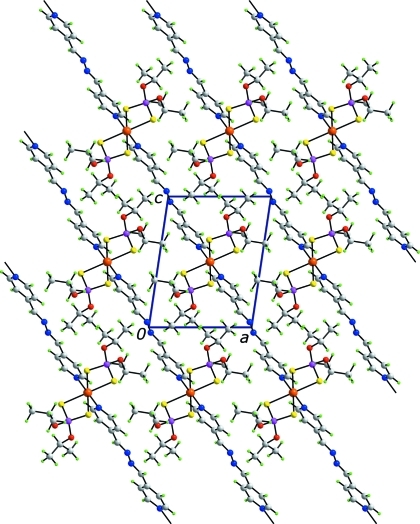
View of the interdigitation of layers in the crystal structure of (I), viewed in projection down the b axis.

## supplementary crystallographic information

### Comment

Interest in molecules related to the title compound (I) revolve around intriguing crystal engineering possibilities whereby different supramolecular topologies may be constructed by careful choice of organic substituents and bridging ligands (Lai & Tiekink, 2004; Chen *et al.*, 2006; Aragoni *et al.*, 2007). The Ni atom in (I), Fig. 1, lies on a crystallographic centre of inversion and the bis(4-pyridylmethylene)diazane molecule is similarly disposed about a centre of inversion. The Ni atom exists within an octahedral *trans*-N_2_S_4_ donor set defined by two symmetrically chelating dithiophosphate ligands and two *trans*-disposed pyridine-N atoms, Table 1. The bridging ligands lead to a linear polymer, Fig. 2, and these are arranged into layers, being connected by C—H···S contacts, Table 2 and Fig. 3. Layers interdigitate to consolidate the crystal packing, Fig. 4.

A similar coordination geometry and linear supramolecular polymer were observed in the iso-butyl derivative of (I) which was characterized crystallographically as a di-toluene solvate (Berdugo & Tiekink, 2008).

### Experimental

Compound (I) was prepared by refluxing the parent nickel dithiophosphate with bis(4-pyridylmethylene)diazane, following a literature procedure (Berdugo & Tiekink, 2008).

### Refinement

The H atoms were geometrically placed (C—H = 0.95–1.00 Å) and refined as riding with *U*_iso_(H) = 1.2*U*_eq_(C) or 1.5*U*_eq_(methyl-C).

### Crystal data

**Table:**

[Ni(C_6_H_14_O_2_PS_2_)_2_(C_12_H_10_N_4_)]	*Z* = 1
*M**_r_* = 695.47	*F*(000) = 364
Triclinic, *P*1	*D*_x_ = 1.381 Mg m^−^^3^
Hall symbol: -P 1	Mo *K*α radiation, λ = 0.71070 Å
*a* = 8.661 (2) Å	Cell parameters from 2817 reflections
*b* = 8.753 (2) Å	θ = 3.0–32.3°
*c* = 11.159 (3) Å	µ = 0.96 mm^−^^1^
α = 88.110 (8)°	*T* = 98 K
β = 81.502 (7)°	Prism, brown-orange
γ = 89.813 (10)°	0.50 × 0.08 × 0.05 mm
*V* = 836.2 (4) Å^3^	

### Data collection

**Table:**

Rigaku AFC12K/SATURN724 diffractometer	3810 independent reflections
Radiation source: fine-focus sealed tube	3555 reflections with *I* > 2σ(*I*)
graphite	*R*_int_ = 0.036
ω scans	θ_max_ = 27.5°, θ_min_ = 2.8°
Absorption correction: multi-scan (*ABSCOR*; Higashi, 1995)	*h* = −9→11
*T*_min_ = 0.794, *T*_max_ = 1	*k* = −11→11
7552 measured reflections	*l* = −14→14

### Refinement

**Table:**

Refinement on *F*^2^	Primary atom site location: structure-invariant direct methods
Least-squares matrix: full	Secondary atom site location: difference Fourier map
*R*[*F*^2^ > 2σ(*F*^2^)] = 0.043	Hydrogen site location: inferred from neighbouring sites
*wR*(*F*^2^) = 0.111	H-atom parameters constrained
*S* = 1.09	*w* = 1/[σ^2^(*F*_o_^2^) + (0.0532*P*)^2^ + 0.6278*P*] where *P* = (*F*_o_^2^ + 2*F*_c_^2^)/3
3810 reflections	(Δ/σ)_max_ < 0.001
178 parameters	Δρ_max_ = 0.63 e Å^−^^3^
0 restraints	Δρ_min_ = −0.70 e Å^−^^3^

### Special details

**Table:**

Geometry. All e.s.d.'s (except the e.s.d. in the dihedral angle between two l.s. planes) are estimated using the full covariance matrix. The cell e.s.d.'s are taken into account individually in the estimation of e.s.d.'s in distances, angles and torsion angles; correlations between e.s.d.'s in cell parameters are only used when they are defined by crystal symmetry. An approximate (isotropic) treatment of cell e.s.d.'s is used for estimating e.s.d.'s involving l.s. planes.
Refinement. Refinement of *F*^2^ against ALL reflections. The weighted *R*-factor *wR* and goodness of fit *S* are based on *F*^2^, conventional *R*-factors *R* are based on *F*, with *F* set to zero for negative *F*^2^. The threshold expression of *F*^2^ > σ(*F*^2^) is used only for calculating *R*-factors(gt) *etc*. and is not relevant to the choice of reflections for refinement. *R*-factors based on *F*^2^ are statistically about twice as large as those based on *F*, and *R*- factors based on ALL data will be even larger.

### Fractional atomic coordinates and isotropic or equivalent isotropic displacement parameters (Å^2^)

**Table:**

	*x*	*y*	*z*	*U*_iso_*/*U*_eq_	
Ni	0.5000	0.5000	0.5000	0.01506 (12)	
S1	0.73976 (6)	0.43573 (6)	0.58844 (5)	0.01900 (14)	
S2	0.46123 (7)	0.68201 (6)	0.66788 (5)	0.01887 (14)	
P1	0.64607 (7)	0.57524 (7)	0.71650 (5)	0.01822 (15)	
O1	0.5953 (2)	0.48949 (19)	0.84272 (15)	0.0223 (4)	
O2	0.7711 (2)	0.6930 (2)	0.75100 (15)	0.0241 (4)	
N1	0.3710 (2)	0.3357 (2)	0.61516 (17)	0.0177 (4)	
N2	0.0259 (2)	0.0611 (2)	0.95949 (18)	0.0230 (4)	
C1	0.6944 (3)	0.3733 (3)	0.8926 (2)	0.0261 (5)	
H1	0.7730	0.3345	0.8257	0.031*	
C2	0.5865 (3)	0.2446 (3)	0.9442 (2)	0.0315 (6)	
H2A	0.5378	0.2009	0.8792	0.047*	
H2B	0.5053	0.2840	1.0061	0.047*	
H2C	0.6464	0.1653	0.9810	0.047*	
C3	0.7778 (3)	0.4463 (3)	0.9858 (2)	0.0305 (6)	
H3A	0.8473	0.5272	0.9464	0.046*	
H3B	0.8393	0.3688	1.0230	0.046*	
H3C	0.7009	0.4903	1.0486	0.046*	
C4	0.8388 (3)	0.8094 (3)	0.6614 (2)	0.0266 (5)	
H4	0.7952	0.7981	0.5840	0.032*	
C5	1.0132 (3)	0.7830 (3)	0.6398 (3)	0.0384 (7)	
H5A	1.0350	0.6822	0.6055	0.058*	
H5B	1.0544	0.7876	0.7168	0.058*	
H5C	1.0634	0.8621	0.5831	0.058*	
C6	0.7934 (3)	0.9641 (3)	0.7138 (3)	0.0380 (7)	
H6A	0.6795	0.9747	0.7249	0.057*	
H6B	0.8410	1.0454	0.6581	0.057*	
H6C	0.8303	0.9718	0.7923	0.057*	
C7	0.4224 (3)	0.1924 (3)	0.6265 (2)	0.0198 (4)	
H7	0.5144	0.1624	0.5755	0.024*	
C8	0.3473 (3)	0.0860 (3)	0.7094 (2)	0.0210 (5)	
H8	0.3865	−0.0153	0.7138	0.025*	
C9	0.2132 (3)	0.1289 (3)	0.7867 (2)	0.0194 (4)	
C10	0.1577 (3)	0.2772 (3)	0.7726 (2)	0.0212 (5)	
H10	0.0653	0.3102	0.8217	0.025*	
C11	0.2384 (3)	0.3754 (3)	0.6868 (2)	0.0208 (5)	
H11	0.1990	0.4758	0.6775	0.025*	
C12	0.1396 (3)	0.0206 (3)	0.8798 (2)	0.0225 (5)	
H12	0.1765	−0.0817	0.8815	0.027*	

### Atomic displacement parameters (Å^2^)

**Table:**

	*U*^11^	*U*^22^	*U*^33^	*U*^12^	*U*^13^	*U*^23^
Ni	0.0173 (2)	0.0127 (2)	0.0143 (2)	0.00009 (15)	0.00006 (15)	0.00119 (14)
S1	0.0197 (3)	0.0198 (3)	0.0170 (3)	0.0034 (2)	−0.0009 (2)	−0.0008 (2)
S2	0.0208 (3)	0.0174 (3)	0.0183 (3)	0.0039 (2)	−0.0023 (2)	−0.0019 (2)
P1	0.0192 (3)	0.0193 (3)	0.0158 (3)	0.0020 (2)	−0.0015 (2)	−0.0011 (2)
O1	0.0243 (8)	0.0249 (8)	0.0174 (8)	0.0055 (7)	−0.0025 (6)	0.0016 (6)
O2	0.0240 (9)	0.0279 (9)	0.0209 (9)	−0.0010 (7)	−0.0045 (7)	−0.0036 (7)
N1	0.0182 (9)	0.0170 (9)	0.0165 (9)	−0.0004 (7)	0.0013 (7)	0.0017 (7)
N2	0.0238 (10)	0.0212 (10)	0.0223 (11)	−0.0065 (8)	0.0006 (8)	0.0090 (8)
C1	0.0270 (12)	0.0323 (13)	0.0186 (12)	0.0112 (10)	−0.0032 (10)	0.0033 (9)
C2	0.0412 (15)	0.0265 (13)	0.0280 (14)	0.0055 (11)	−0.0096 (11)	0.0026 (10)
C3	0.0256 (13)	0.0412 (15)	0.0250 (13)	0.0024 (11)	−0.0059 (10)	0.0051 (11)
C4	0.0274 (12)	0.0259 (12)	0.0266 (13)	−0.0038 (10)	−0.0034 (10)	−0.0033 (10)
C5	0.0279 (14)	0.0344 (15)	0.0503 (19)	−0.0032 (12)	0.0044 (12)	−0.0087 (13)
C6	0.0298 (14)	0.0289 (14)	0.056 (2)	0.0019 (11)	−0.0081 (13)	−0.0088 (13)
C7	0.0222 (11)	0.0181 (10)	0.0184 (11)	0.0002 (9)	−0.0008 (9)	0.0019 (8)
C8	0.0240 (11)	0.0155 (10)	0.0233 (12)	0.0003 (9)	−0.0028 (9)	0.0013 (8)
C9	0.0193 (11)	0.0199 (11)	0.0191 (11)	−0.0027 (8)	−0.0040 (8)	0.0028 (8)
C10	0.0198 (11)	0.0209 (11)	0.0210 (12)	−0.0004 (9)	0.0023 (9)	0.0019 (9)
C11	0.0209 (11)	0.0184 (10)	0.0213 (12)	0.0023 (9)	0.0020 (9)	0.0049 (8)
C12	0.0229 (11)	0.0224 (11)	0.0228 (12)	−0.0046 (9)	−0.0062 (9)	0.0056 (9)

### Geometric parameters (Å, °)

**Table:**

Ni—S1	2.4827 (7)	C3—H3A	0.9800
Ni—S2	2.4835 (7)	C3—H3B	0.9800
Ni—N1	2.1051 (19)	C3—H3C	0.9800
Ni—N1^i^	2.1051 (19)	C4—C5	1.513 (4)
Ni—S1^i^	2.4827 (7)	C4—C6	1.520 (4)
Ni—S2^i^	2.4835 (7)	C4—H4	1.0000
S1—P1	1.9895 (9)	C5—H5A	0.9800
S2—P1	1.9859 (9)	C5—H5B	0.9800
P1—O1	1.5779 (17)	C5—H5C	0.9800
P1—O2	1.5934 (18)	C6—H6A	0.9800
O1—C1	1.476 (3)	C6—H6B	0.9800
O2—C4	1.464 (3)	C6—H6C	0.9800
N1—C7	1.338 (3)	C7—C8	1.384 (3)
N1—C11	1.350 (3)	C7—H7	0.9500
N2—C12	1.283 (3)	C8—C9	1.399 (3)
N2—N2^ii^	1.408 (4)	C8—H8	0.9500
C1—C2	1.510 (4)	C9—C10	1.395 (3)
C1—C3	1.511 (4)	C9—C12	1.459 (3)
C1—H1	1.0000	C10—C11	1.377 (3)
C2—H2A	0.9800	C10—H10	0.9500
C2—H2B	0.9800	C11—H11	0.9500
C2—H2C	0.9800	C12—H12	0.9500
			
N1—Ni—S1^i^	89.02 (6)	C1—C3—H3B	109.5
N1^i^—Ni—S1^i^	90.98 (6)	H3A—C3—H3B	109.5
N1—Ni—S1	90.98 (6)	C1—C3—H3C	109.5
N1^i^—Ni—S1	89.02 (6)	H3A—C3—H3C	109.5
N1—Ni—S2^i^	91.02 (6)	H3B—C3—H3C	109.5
N1^i^—Ni—S2^i^	88.98 (6)	O2—C4—C5	107.1 (2)
S1^i^—Ni—S2^i^	82.46 (2)	O2—C4—C6	107.0 (2)
S1—Ni—S2^i^	97.54 (2)	C5—C4—C6	113.4 (2)
N1—Ni—S2	88.98 (6)	O2—C4—H4	109.8
N1^i^—Ni—S2	91.02 (6)	C5—C4—H4	109.8
S1^i^—Ni—S2	97.54 (2)	C6—C4—H4	109.8
S1—Ni—S2	82.46 (2)	C4—C5—H5A	109.5
S1^i^—Ni—S1	180.0	C4—C5—H5B	109.5
S2^i^—Ni—S2	180.0	H5A—C5—H5B	109.5
N1^i^—Ni—N1	180.0	C4—C5—H5C	109.5
P1—S1—Ni	82.80 (3)	H5A—C5—H5C	109.5
P1—S2—Ni	82.85 (3)	H5B—C5—H5C	109.5
O1—P1—O2	100.84 (10)	C4—C6—H6A	109.5
O1—P1—S2	108.61 (7)	C4—C6—H6B	109.5
O2—P1—S2	111.61 (7)	H6A—C6—H6B	109.5
O1—P1—S1	112.72 (7)	C4—C6—H6C	109.5
O2—P1—S1	111.82 (7)	H6A—C6—H6C	109.5
S2—P1—S1	110.84 (4)	H6B—C6—H6C	109.5
C1—O1—P1	122.47 (15)	N1—C7—C8	122.9 (2)
C4—O2—P1	119.75 (15)	N1—C7—H7	118.6
C7—N1—C11	117.73 (19)	C8—C7—H7	118.6
C7—N1—Ni	121.63 (15)	C7—C8—C9	119.4 (2)
C11—N1—Ni	120.48 (15)	C7—C8—H8	120.3
C12—N2—N2^ii^	111.3 (2)	C9—C8—H8	120.3
O1—C1—C2	106.2 (2)	C10—C9—C8	117.6 (2)
O1—C1—C3	108.7 (2)	C10—C9—C12	122.7 (2)
C2—C1—C3	113.5 (2)	C8—C9—C12	119.7 (2)
O1—C1—H1	109.5	C11—C10—C9	119.2 (2)
C2—C1—H1	109.5	C11—C10—H10	120.4
C3—C1—H1	109.5	C9—C10—H10	120.4
C1—C2—H2A	109.5	N1—C11—C10	123.1 (2)
C1—C2—H2B	109.5	N1—C11—H11	118.4
H2A—C2—H2B	109.5	C10—C11—H11	118.4
C1—C2—H2C	109.5	N2—C12—C9	121.1 (2)
H2A—C2—H2C	109.5	N2—C12—H12	119.4
H2B—C2—H2C	109.5	C9—C12—H12	119.4
C1—C3—H3A	109.5		
			
N1—Ni—S1—P1	−82.10 (6)	S2—Ni—N1—C7	−131.96 (18)
N1^i^—Ni—S1—P1	97.90 (6)	S1^i^—Ni—N1—C11	−54.34 (18)
S2^i^—Ni—S1—P1	−173.26 (3)	S1—Ni—N1—C11	125.66 (18)
S2—Ni—S1—P1	6.74 (3)	S2^i^—Ni—N1—C11	−136.78 (18)
N1—Ni—S2—P1	84.37 (6)	S2—Ni—N1—C11	43.22 (18)
N1^i^—Ni—S2—P1	−95.63 (6)	P1—O1—C1—C2	−137.26 (18)
S1^i^—Ni—S2—P1	173.24 (3)	P1—O1—C1—C3	100.3 (2)
S1—Ni—S2—P1	−6.76 (3)	P1—O2—C4—C5	−119.8 (2)
Ni—S2—P1—O1	−115.40 (7)	P1—O2—C4—C6	118.37 (19)
Ni—S2—P1—O2	134.30 (7)	C11—N1—C7—C8	−1.3 (3)
Ni—S2—P1—S1	8.96 (3)	Ni—N1—C7—C8	174.02 (18)
Ni—S1—P1—O1	113.02 (8)	N1—C7—C8—C9	−1.1 (4)
Ni—S1—P1—O2	−134.19 (7)	C7—C8—C9—C10	2.6 (3)
Ni—S1—P1—S2	−8.96 (3)	C7—C8—C9—C12	−175.8 (2)
O2—P1—O1—C1	−75.87 (19)	C8—C9—C10—C11	−1.8 (3)
S2—P1—O1—C1	166.73 (16)	C12—C9—C10—C11	176.6 (2)
S1—P1—O1—C1	43.51 (19)	C7—N1—C11—C10	2.1 (4)
O1—P1—O2—C4	−173.76 (16)	Ni—N1—C11—C10	−173.22 (18)
S2—P1—O2—C4	−58.58 (17)	C9—C10—C11—N1	−0.6 (4)
S1—P1—O2—C4	66.22 (17)	N2^ii^—N2—C12—C9	−179.5 (2)
S1^i^—Ni—N1—C7	130.48 (18)	C10—C9—C12—N2	−5.2 (4)
S1—Ni—N1—C7	−49.52 (18)	C8—C9—C12—N2	173.1 (2)
S2^i^—Ni—N1—C7	48.04 (18)		

Symmetry codes: (i) −*x*+1, −*y*+1, −*z*+1; (ii) −*x*, −*y*, −*z*+2.

### Hydrogen-bond geometry (Å, °)

**Table:**

*D*—H···*A*	*D*—H	H···*A*	*D*···*A*	*D*—H···*A*
C8—H8···S2^iii^	0.95	2.77	3.694 (3)	164

Symmetry codes: (iii) *x*, *y*−1, *z*.
